# Biochar enhances seed germination and crop early growth for sustainable agriculture in Bangladesh

**DOI:** 10.1371/journal.pone.0320005

**Published:** 2025-03-18

**Authors:** Md Rezaul Karim, Sonchita Biswas, Md Abdul Halim, Romel Ahmed

**Affiliations:** 1 Institute of Forestry and Conservation, John H Daniels Faculty of Architecture Landscape and Design, University of Toronto, Toronto, Ontario, Canada; 2 Department of Forestry and Environmental Science, Shahjalal University of Science and Technology, Sylhet, Bangladesh; 3 Center for Research in Environment, iGen and Livelihood (CREGL), Sylhet, Bangladesh; Government College University Faisalabad, Pakistan, PAKISTAN

## Abstract

Biochar (BC) application to low-fertility soils enhances crop yield, soil quality, and sustainable agricultural production. Although many studies have explored the effects of biochar on tropical crops, research specific to Bangladesh is limited. Given the agrarian system in Bangladesh, dense population, and vulnerability to climate change, adopting sustainable agricultural practices is essential. This study evaluates the impact of different biochar dosages on the germination and early growth of five major crops *Oryza sativa* (rice), *Triticum aestivum* (wheat), *Capsicum annuum* (chili), *Solanum melongena* (eggplant), and *Solanum lycopersicum* (tomato) using *Acacia auriculiformis* wood-waste biochar. The research was conducted using a randomized complete block design (RCBD) in a nursery setting. Biochar treatments of 10 t/ha and 15 t/ha were applied, with assessments made of germination (%), germination rate (after 7 days), shoot height (cm), root height (cm), leaf number, and root-shoot dry weight ratio. The results indicated a significant (p < 0.001) increase in germination (%) with higher biochar application rates. The linear mixed-effects model showed a significant effect of biochar treatment on germination (%) (F = 57.33, p < 0.001) and a significant interaction with crop type (F = 15.84, p < 0.001). In *C. annuum*, the 15 t/ha treatment resulted in a 96% increase in germination compared to the control (43.3 ± 1.08% vs 85.1 ± 2.15%). Similarly, in *O. sativa*, germination was significantly higher with the 10 t/ha (84.5 ± 1.52%) and 15 t/ha (91.8 ± 1.49%) treatments compared to control (59.3 ± 2.38%). Biochar significantly (p < 0.05) influenced early germination rates (after 7 days) and early growth parameters (e.g., shoot length, leaf count, root-shoot ratio), with the 15 t/ha treatment showing substantial improvements for *C. annuum* and *O. sativa*, while no significant effects were observed for *S. lycopersicum*. These findings underscore the potential of *A. auriculiformis* in enhancing germination and early growth of economically important crops, highlighting its role in promoting sustainable agriculture in Bangladesh.

## Introduction

Agriculture in tropical regions faces significant challenges due to land degradation and environmental pollution, exacerbated by the pressures of population growth and the rapid changes induced by climate change [1]. This challenge is particularly severe in developing countries, such as Bangladesh, where high population density and shrinking arable land make it challenging to meet the food demand of the population [2]. Furthermore, climate change compounds these challenges by altering precipitation patterns, increasing the frequency of extreme weather events, and introducing new stresses on agriculture [3]. The high demand for short-lifecycle crops, compared to perennial crops, further exacerbates concerns about the need for high productivity [4]. Additionally, various stresses and challenges in tropical regions result in reduced seed germination rates, thereby threatening food security [5,6]. Therefore, ensuring high germination potential and healthy early growth of tropical crops is imperative.

Agriculture in Bangladesh faces immense pressure to increase productivity while maintaining environmental sustainability. Agriculture in the country is predominantly characterized by the cultivation of staple crops, including several varieties of rice (*Oryza sativa*), wheat (*Triticum aestivum*), potato (*Solanum tuberosum*), lentil (*Vicia lens* (L.) Coss. & Germ), chili (*Capsicum annuum*), tomato (*Solanum lycopersicum*), and eggplant (*Solanum melongena*). However, crop productivity is severely threatened by soil degradation, nutrient depletion, and water scarcity, all of which are exacerbated by climate change [2].

To address these challenges, crop management techniques, particularly those focused on climate-smart crop management, have acquired significant attention globally [7]. Recent research indicates that biochar, a carbon-rich byproduct obtained from the pyrolysis of biomass, can effectively amend degraded lands [8]. Biochar has emerged as a promising solution due to its capacity to enhance soil quality by improving water retention, increasing nutrient availability, and boosting cation exchange capacity of the soil [9,10].

These improvements can lead to better seed germination, plant growth, and crop yields, particularly in tropical regions where soil fertility is often low [11]. For example, various doses of woody biochar (primarily 10 t/ha) positively affect the germination rate and seedling growth of rice (*Oryza sativa*) seeds compared to control or raw feedstock treatments in tropical environments [12]. Moreover, biochar application at different concentrations (>50t/ha) has been shown to promote wheat germination [13]. Biochar can be applied alongside organic or inorganic fertilizers, significantly enhancing crop yield [14]. However, some biochar formulations may contain substances that adversely affect seed germination and early seedling growth [15]. Therefore, it is imperative to assess the impact of any biochar on these parameters prior to its large-scale application in major crops within the agricultural systems of Bangladesh and similar tropical regions.

Despite the potential benefits of biochar, research on biochar application on major crops in Bangladesh remains largely unexplored [12]. Understanding its influence on germination and early growth stages is crucial for developing sustainable agricultural practices that ensure food security amidst climate change. Biochar produced from woody residues significantly enhances agricultural productivity, making it a practical choice for soil amendment [16,17]. Furthermore, biochar application reduces nutrient leaching, supports microbial populations, and suppresses pests [18]. It can also mitigate plant stresses associated with drought and exposure to toxic materials. Additionally, biochar can absorb a wide range of potentially toxic materials, including heavy metals, pesticides, and other contaminants [19]. Moreover, biochar application has been shown to increase both above- and below-ground biomass, plant height, and leaf number in comparison to control without the biochar [20].

In Bangladesh, where scarcity of irrigation water and nutrient depletion are major issues, biochar can play a crucial role in improving water use efficiency [9] and nutrient retention [10], reducing the need for chemical fertilizers and irrigation. This research aims to evaluate the effects of biochar produced from *Acacia auriculiformis* on the germination and early growth of five widely used agricultural species in the tropics and staple crops in Bangladesh: rice (*Oryza sativa)*, wheat (*Triticum aestivum)*, chili (*Capsicum annuum)*, tomato (*Solanum lycopersicum)*, and eggplant (*Solanum melongena)*. Specifically, the study aims to: a) assess the effect of *Acacia auriculiformis* biochar on the seed germination of these species, and b) evaluate its impact on early seedling growth, including shoot length, root length, leaf number, and root-to-shoot weight ratio.

This research will contribute to sustainable agricultural practices, addressing the challenges of land degradation, climate change, and food security. By providing insights into the benefits of biochar for major crops, this study aims to support the adoption of sustainable agricultural practices that can enhance productivity and resilience in tropical agriculture.

## Materials and methods

### Study area

The experiment was conducted at the nursery of the Department of Forestry and Environmental Science, Shahjalal University of Science and Technology (SUST), Sylhet, Bangladesh (latitude: 24° 55’ 15.56“, longitude: 91° 50’ 4.49”). The climate of the SUST region is characterized as a humid subtropical. The average maximum temperature ranges around 23°C from August to October, while the average minimum temperature is approximately 7°C in January. The region experiences an annual average rainfall of 3,334 mm, with 80% of the total precipitation occurring between May and September [9].

### Test crop and feedstock species

Five widely cultivated crops in the tropics were selected for this study: rice, wheat, chili, tomato, and eggplant (Table 1).

**Table 1:** Test agricultural and feedstock species used in the study.

Name		Scientific name	Family
Test crops	Rice	*Oryza sativa* L. var. BR32	*Poaceae*
	Wheat	*Triticum aestivum* L.	*Poaceae*
	Eggplant	*Solanum melongena* L.	*Solanaceae*
	Chili	*Capsicum annuum* L.	*Solanaceae*
	Tomato	*Solanum lycopersicum* L.	*Solanaceae*
Feedstock	Acacia	*Acacia auriculiformis* A.Cunn. ex Benth.	*Fabaceae*

These crops were chosen as they are the main crops in Bangladesh, have relatively short growth cycles, reaching maturity and productivity within 3 to 4 months, and their potential for high yield under optimal management conditions.

### Biochar production and characterization of physiochemical properties

The biochar used in this study was produced following the same methodology described in our previous work [9], using a locally-built pyrolysis technology. The feedstock consisted of offcuts from a small sawmill processing *Acacia auriculiformis* A.Cunn. ex Benth., a commonly planted legume. A low-tech, open conical “flame curtain” kiln, similar to the “Kon Tiki” kiln [21], was used in pyrolysis. The pyrolysis process typically lasted around 3.5 hours, with temperatures maintaining an average of 450 °C and peaking at up to 550 °C. Immediately after removal from the kiln, the biochar was washed with water for 30 minutes to reduce low molecular weight organic compounds [22] and then sun-dried for three days before being applied to the soil surface. Physicochemical analyses of the biochar were conducted following the protocol described in Karim et al. (2020). Elemental analyses for total carbon (C) and nitrogen (N) were performed using a LECO 628 CN analyzer. Trace elements were quantified following a four-acid digestion procedure, subsequently analyzed by inductively coupled plasma mass spectrometry (ICP-MS) at Activation Laboratories Ltd. (Ancaster, ON, Canada). The assessments of volatile matter, ash content, pH, electrical conductivity, and bulk density were conducted in accordance with the standardized methodologies previously described [9]. Detailed physiochemical properties of the biochar are provided in S1 File.

### Soil collection and pot filling

Soil samples were collected from a fallow land in the vicinity of Shahjalal University of Science and Technology (SUST), Sylhet, Bangladesh. The soil predominantly exhibited a sandy loam texture and had not been subjected to fertilization treatments. Prior to experimentation, the soil samples underwent meticulous sieving to eliminate extraneous debris or roots, ensuring uniformity. A total of 45 pots (surface area 230 cm²) were designated for the germination phase of the experiment, while 75 pots were allocated for the early growth stage. Subsequently, each pot was uniformly filled with 2 kg of soil, maintaining consistency across experimental conditions.

### Treatments

Three experimental treatments were established, consisting of biochar application rates of 10 t/ha and 15 t/ha, along with a control group receiving no biochar. Each treatment was replicated three times during the germination phase and five times for the growth performance evaluation phase. For biochar application, 23 g and 34.5 g were respectively applied to the surface of each pot, corresponding to the 10 t/ha and 15 t/ha treatment rates. The selected treatment levels are consistent with those used in previous studies in the tropics [23,24].

### Experimental design

A randomized complete block design (RCBD) was used for this study. During the germination phase, three treatments (control, 10 t/ha, and 15 t/ha) were replicated three times for each crop species, resulting in a total of 45 pots (5 species ×  3 treatments ×  3 replications). Each pot was sown with three seeds of the respective species, totaling 135 seeds across all treatments and replications. In the growth phase, the number of replications per treatment was increased to five for each crop species to facilitate a more robust assessment of early seedling growth. As a result, the total number of pots was increased to 75 (5 species ×  3 treatments ×  5 replications). These 75 pots were randomized across five blocks, with each block containing 15 pots (3 treatments ×  5 species).

### Germination and early growth parameters

Daily observations were conducted to record the number of germinated seeds. Following germination, data on early growth parameters, such as shoot length, root length, leaf number, and other pertinent variables, were systematically collected for a duration of 40 days for final data collection, while the first data was collected after 7 days for the first germination percentage. Upon reaching maturity after 120 days, the crops were harvested, and measurements of fresh and dry weights for both shoots and roots were obtained.

### Data analysis

#### Germination parameter estimation.

Upon seed sowing, the emergence of seedlings was meticulously recorded daily until the completion of germination [25]. The following equations were utilized to calculate the germination percentage:


$$Germination\left(\%\right)=\frac{\text{Total number seeds germinated after }40\text{days early growth period}}{\text{Total seeds }}\times 100$$

The germination rate for early responses of the crops was assessed after seven days. This initial germination rate is crucial for evaluating early establishment and viability. A rapid germination rate suggests favorable conditions for seedling development, which can significantly impact crop yield and productivity.

The germination rate after seven days was determined using the following equation:


$$Rateofgermination=\frac{\text{Total number seeds germinated after first }7\text{days }}{\text{Total seeds }}\times 100$$

#### Early growth measurement.

To assess the early growth of seedlings, shoot length measurements were obtained using a tape measure at 7-day intervals. Additionally, the primary root lengths of each seedling were measured at the end of the experiment. The number of leaves was also counted, and the stems of individual seedlings were cut into small sections and dried in an oven at 65°C for 48 hours in paper bags. Subsequently, their dry weights were recorded. Similarly, the roots of individual seedlings were meticulously washed, dried under the same conditions, and weighed to determine the dry weight root-to-shoot ratio.

### Statistical analysis

To test the effects of biochar on crop germination and early growth, we employed linear mixed-effects models performed using the *lme()* function of the “*nlme*” package [26] in the R version 4.4.1 [27]. Initially, we considered root and shoot length, leaf count, root-to-shoot dry weight ratio, germination (%), and germination rate (raw values) as dependent variables, with treatments (biochar and crop) as fixed effects. Replicated samples were nested within each block, and blocks were nested within the number of measurements (to account for repeated measures) as the random effect in these models. To assess the effect of biochar, we performed ANOVAs (with likelihood comparison of fitted models) on the linear mixed-effects models of plant germination and early growth properties throughout the experiment. For multiple comparisons of crop ×  biochar combinations, we compared estimated marginal means using the *emmeans*() and *contrast*() (with method =  “pairwise”) functions of the R-package “*emmeans*” [28]. The residual normality of linear mixed-effects models was assessed graphically using histograms and Q-Q probability plots and was found to be normal for all models.

## Result

### Biochar effects on early growth germination (%) and rate of germination (after 7 days)

**Fig 1:**
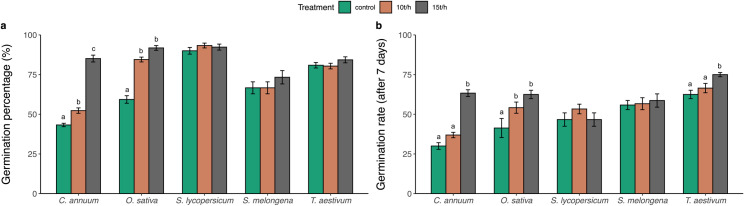
a) Germination percentage and b) Germination rates after a seven-day growth period for five crops species.

The application of biochar significantly affected the germination (%) across different crops (Fig 1a; Table 2).

**Table 2:** Results of linear mixed-effects models examining biochar effects on various treatments by crops. Statistical significance: p < 0.05 (*), p < 0.01 (**), and p < 0.001 (***).

Factor	Response	Df	F value	Pr(>F)
Treatment	Root length	2	0.747	0.48
Treatment: Crop	Root length	8	4.923	<0.001^***^
Treatment	Shoot length	2	5.026	<0.05^ * ^
Treatment: Crop	Shoot length	8	0.596	0.7751
Treatment	Leaf count	2	8.318	<0.001^***^
Treatment: Crop	Leaf count	8	5.136	<0.001^***^
Treatment	Root shoot weight ratio	2	8.764	<0.001^***^
Treatment: Crop	Root shoot weight ratio	8	6.532	<0.001^***^
Treatment	Germination (%)	2	57.33	<0.001^***^
Treatment: Crop	Germination (%)	8	15.84	<0.001^***^
Treatment	Rate of germination	2	25.18	<0.001^***^
Treatment: Crop	Rate of germination	8	6.07	<0.001^***^

The linear mixed-effects model (LMM) analysis revealed a significant effect of biochar treatment on germination (%) (F = 57.33, p < 0.001), and a significant interaction was observed between treatment and crop type (F = 15.84, p < 0.001) on germination (%).

In *C. annuum*, biochar treatments significantly enhanced germination (%) compared to the control, with the 15 t/ha treatment resulting in a 96% improvement (control: 43.3 ± 1.08%, 10 t/ha: 52.3 ± 1.70%, 15 t/ha: 85.1 ± 2.15%). Similarly, in *O. sativa*, germination (%) was notably higher with biochar application (control: 59.3 ± 2.38%, 10 t/ha: 84.5 ± 1.52%, 15 t/ha: 91.8 ± 1.49%), however, no further significant increase was observed beyond the 10 t/ha treatment (p = 0.12). In contrast, biochar did not produce significant changes in germination (%) for *S. lycopersicum* (control: 90 ± 2.11%, 10 t/ha: 93.3 ± 1.52%, 15 t/ha: 92.3 ± 1.89%), *S. melongena* (control: 66.7 ± 3.77%, 10 t/ha: 66.7 ± 3.77%, 15 t/ha: 73.3 ± 4.22%), or *T. aestivum* (control: 80.9 ± 1.72%, 10 t/ha: 80.4 ± 1.77%, 15 t/ha: 84.3 ± 1.89%).

Furthermore, biochar application significantly influenced early germination rates (after 7 days) (Fig 1b; Table 3), with both treatment effects (F = 25.18, p < 0.001) and an interaction with crop type (F = 6.07, p < 0.001).

**Table 3:** Post hoc Tukey comparison among several crops with germination and early growth responses. Statistical significance: p < 0.05 (*), p < 0.01 (**), and p < 0.001 (***).

Response	Crop	Treatment	Estimate	SE	df	t.ratio	p.value
Root length (cm)	*C. annuum*	control - (10t/h)	−1.432	0.756	40	−1.893	0.1538
	*C. annuum*	control - (15t/h)	−1.420	0.756	40	−1.877	0.1585
	*C. annuum*	(10t/h) - (15t/h)	0.012	0.756	40	0.016	0.9999
	*O. sativa*	control - (10t/h)	−1.360	0.756	40	−1.798	0.1832
	*O. sativa*	control - (15t/h)	−2.800	0.756	40	−3.702	0.0018^**^
	*O. sativa*	(10t/h) - (15t/h)	−1.440	0.756	40	−1.904	0.1508
	*S. lycopersicum*	control - (10t/h)	0.680	0.756	40	0.899	0.6441
	*S. lycopersicum*	control - (15t/h)	3.020	0.756	40	3.993	0.0008^***^
	*S. lycopersicum*	(10t/h) - (15t/h)	2.340	0.756	40	3.094	0.0099^**^
	*S. melongena*	control - (10t/h)	−0.840	0.756	40	−1.111	0.5133
	*S. melongena*	control - (15t/h)	−1.000	0.756	40	−1.322	0.3915
	*S. melongena*	(10t/h) - (15t/h)	−0.160	0.756	40	−0.212	0.9757
	*T. aestivum*	control - (10t/h)	1.240	0.756	40	1.639	0.2412
	*T. aestivum*	control - (15t/h)	0.340	0.756	40	0.449	0.8949
	*T. aestivum*	(10t/h) - (15t/h)	−0.900	0.756	40	−1.190	0.4661
Shoot length (cm)	*C. annuum*	control - (10t/h)	−3.40	1.48	40	−2.294	0.0682
	*C. annuum*	control - (15t/h)	−1.04	1.48	40	−0.702	0.7639
	*C. annuum*	(10t/h) - (15t/h)	2.36	1.48	40	1.592	0.2607
	*O. sativa*	control - (10t/h)	−2.28	1.48	40	−1.538	0.2842
	*O. sativa*	control - (15t/h)	−1.14	1.48	40	−0.769	0.7239
	*O. sativa*	(10t/h) - (15t/h)	1.14	1.48	40	0.769	0.7239
	*S. lycopersicum*	control - (10t/h)	−2.50	1.48	40	−1.687	0.2227
	*S. lycopersicum*	control - (15t/h)	−1.00	1.48	40	−0.675	0.7794
	*S. lycopersicum*	(10t/h) - (15t/h)	1.50	1.48	40	1.012	0.5737
	*S. melongena*	control - (10t/h)	0.14	1.48	40	0.094	0.9951
	*S. melongena*	control - (15t/h)	1.36	1.48	40	0.918	0.6325
	*S. melongena*	(10t/h) - (15t/h)	1.22	1.48	40	0.823	0.6910
	*T. aestivum*	control - (10t/h)	−2.36	1.48	40	−1.592	0.2607
	*T. aestivum*	control - (15t/h)	−2.08	1.48	40	−1.403	0.3488
	*T. aestivum*	(10t/h) - (15t/h)	0.28	1.48	40	0.189	0.9805
Leaf count	*C. annuum*	control - (10t/h)	−1.0	0.36	40	−2.781	0.0220^ * ^
	*C. annuum*	control - (15t/h)	−0.8	0.36	40	−2.225	0.0792
	*C. annuum*	(10t/h) - (15t/h)	0.2	0.36	40	0.556	0.8439
	*O. sativa*	control - (10t/h)	−0.6	0.36	40	−1.668	0.2298
	*O. sativa*	control - (15t/h)	−1.0	0.36	40	−2.781	0.0220^ * ^
	*O. sativa*	(10t/h) - (15t/h)	−0.4	0.36	40	−1.112	0.5122
	*S. lycopersicum*	control - (10t/h)	−1.2	0.36	40	−3.337	0.0051^**^
	*S. lycopersicum*	control - (15t/h)	−1.0	0.36	40	−2.781	0.0220^ * ^
	*S. lycopersicum*	(10t/h) - (15t/h)	0.2	0.36	40	0.556	0.8439
	*S. melongena*	control - (10t/h)	−0.4	0.36	40	−1.112	0.5122
	*S. melongena*	control - (15t/h)	0.8	0.36	40	2.225	0.0792
	*S. melongena*	(10t/h) - (15t/h)	1.2	0.36	40	3.337	0.0051^**^
	*T. aestivum*	control - (10t/h)	0.6	0.36	40	1.668	0.2298
	*T. aestivum*	control - (15t/h)	−0.4	0.36	40	−1.112	0.5122
	*T. aestivum*	(10t/h) - (15t/h)	−1.0	0.36	40	−2.781	0.0220^ * ^
Root shoot weight ratio	*C. annuum*	control - (10t/h)	0.16690	0.199	40	0.840	0.6805
	*C. annuum*	control - (15t/h)	0.41216	0.199	40	2.075	0.1080
	*C. annuum*	(10t/h) - (15t/h)	0.24526	0.199	40	1.235	0.4401
	*O. sativa*	control - (10t/h)	1.25246	0.199	40	6.305	<.0001^***^
	*O. sativa*	control - (15t/h)	1.28969	0.199	40	6.492	<.0001^***^
	*O. sativa*	(10t/h) - (15t/h)	0.03724	0.199	40	0.187	0.9808
	*S. lycopersicum*	control - (10t/h)	−0.01576	0.199	40	−0.079	0.9965
	*S. lycopersicum*	control - (15t/h)	−0.50738	0.199	40	−2.554	0.0379^ * ^
	*S. lycopersicum*	(10t/h) - (15t/h)	−0.49162	0.199	40	−2.475	0.0455^ * ^
	*S. melongena*	control - (10t/h)	0.10764	0.199	40	0.542	0.8512
	*S. melongena*	control - (15t/h)	−0.00967	0.199	40	−0.049	0.9987
	*S. melongena*	(10t/h) - (15t/h)	−0.11731	0.199	40	−0.591	0.8260
	*T. aestivum*	control - (10t/h)	0.23403	0.199	40	1.178	0.4729
	*T. aestivum*	control - (15t/h)	0.24380	0.199	40	1.227	0.4443
	*T. aestivum*	(10t/h) - (15t/h)	0.00977	0.199	40	0.049	0.9987
Germination (%)	*C. annuum*	control - (10t/h)	−9.043	3.63	40	−2.488	0.0442^ * ^
	*C. annuum*	control - (15t/h)	−41.843	3.63	40	−11.511	<.0001^***^
	*C. annuum*	(10t/h) - (15t/h)	−32.800	3.63	40	−9.024	<.0001^***^
	*O. sativa*	control - (10t/h)	−25.171	3.63	40	−6.925	<.0001^***^
	*O. sativa*	control - (15t/h)	−32.485	3.63	40	−8.937	<.0001^***^
	*O. sativa*	(10t/h) - (15t/h)	−7.313	3.63	40	−2.012	0.1224
	*S. lycopersicum*	control - (10t/h)	−3.333	3.63	40	−0.917	0.6328
	*S. lycopersicum*	control - (15t/h)	−2.333	3.63	40	−0.642	0.7980
	*S. lycopersicum*	(10t/h) - (15t/h)	1.000	3.63	40	0.275	0.9592
	*S. melongena*	control - (10t/h)	0.000	3.63	40	0.000	1.0000
	*S. melongena*	control - (15t/h)	−6.667	3.63	40	−1.834	0.1716
	*S. melongena*	(10t/h) - (15t/h)	−6.667	3.63	40	−1.834	0.1716
	*T. aestivum*	control - (10t/h)	0.536	3.63	40	0.147	0.9881
	*T. aestivum*	control - (15t/h)	−3.399	3.63	40	−0.935	0.6217
	*T. aestivum*	(10t/h) - (15t/h)	−3.934	3.63	40	−1.082	0.5304
Germination rate (after 7 days)	*C. annuum*	control - (10t/h)	−6.923	4.41	40	−1.570	0.2702
	*C. annuum*	control - (15t/h)	−33.333	4.41	40	−7.560	<.0001^***^
	*C. annuum*	(10t/h) - (15t/h)	−26.411	4.41	40	−5.990	<.0001^***^
	*O. sativa*	control - (10t/h)	−12.827	4.41	40	−2.909	0.0159^ * ^
	*O. sativa*	control - (15t/h)	−21.161	4.41	40	−4.799	0.0001^***^
	*O. sativa*	(10t/h) - (15t/h)	−8.333	4.41	40	−1.890	0.1547
	*S. lycopersicum*	control - (10t/h)	−6.667	4.41	40	−1.512	0.2962
	*S. lycopersicum*	control - (15t/h)	0.000	4.41	40	0.000	1.0000
	*S. lycopersicum*	(10t/h) - (15t/h)	6.667	4.41	40	1.512	0.2962
	*S. melongena*	control - (10t/h)	−0.838	4.41	40	−0.190	0.9803
	*S. melongena*	control - (15t/h)	−2.838	4.41	40	−0.644	0.7969
	*S. melongena*	(10t/h) - (15t/h)	−2.000	4.41	40	−0.454	0.8931
	*T. aestivum*	control - (10t/h)	−4.000	4.41	40	−0.907	0.6390
	*T. aestivum*	control - (15t/h)	−12.500	4.41	40	−2.835	0.0192^ * ^
	*T. aestivum*	(10t/h) - (15t/h)	−8.500	4.41	40	−1.928	0.1441

In *C. annuum*, the 15 t/ha treatment substantially improved early germination rates (control: 30 ± 2.11%, 10 t/ha: 36.9 ± 1.71%, 15 t/ha: 63.3 ± 2.11%), and similar trends were observed in *O. sativa* (control: 41.3 ± 6.00%, 10 t/ha: 54.2 ± 3.53%, 15 t/ha: 62.5 ± 2.64%). However, no significant (p > 0.05) changes in early germination rates were observed for *S. lycopersicum* (control: 46.7 ± 4.22%, 10 t/h: 53.3 ± 3.03%, 15 t/h: 46.7 ± 4.22%)*, S. melongena* (control: 55.8 ± 2.87%, 10 t/h: 56.7 ± 3.77%, 15 t/h: 58.7 ± 4.19%)*,* and *T. aestivum* (control: 62.5 ± 2.64%, 10 t/h: 66.5 ± 2.93%, 15 t/h: 75 ± 1.33%) (Fig 1b, Table 3).

### Biochar effects on early growth

#### Root length.

**Fig 2:**
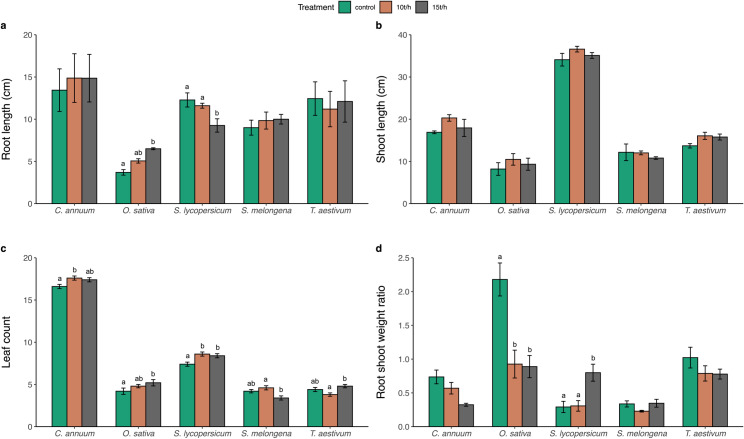
Bar plot comparing a) Root length (cm), b) Shoot length (cm), c) Leaf count, and d) Root shoot dry weight ratio across five crop species.

Biochar treatment did not show a consistent impact on root length (cm) across all crops (Fig 2a; Table 2).

ANOVA results indicated that the effect of biochar treatment on root length was not significant (F = 0.747, p = 0.48) (Table 2). However, the interaction between treatment and crop was significant (F = 4.923, p < 0.001) (Table 2). Significant contrasts (p < 0.01) were observed for *O. sativa* between the control (3.7 ± 0.326 cm) and 15 t/h treatments (6.5 ± 0.114 cm) and for *S. lycopersicum* between the control (12.3 ± 0.836 cm) and 15 t/h treatments (9.26 ± 0.789 cm) (p < 0.001) (Table 3).

#### Shoot length.

The response of shoot length (cm) to biochar treatment varied among the crops (Fig 2b; Table 2). ANOVA results indicated a significant effect of treatment on shoot length (F = 5.026, p < 0.05), while the interaction between treatment and crop was not significant (F = 0.596, p = 0.7751) (Table 2). All crops showed no significant differences (p > 0.05) in shoot length under biochar doses (Fig 2b). The highest shoot length was observed in *S. lycopersicum* with the 10 t/ha treatment (36.6 ± 0.678 cm) and the 15 t/ha treatment (35.1 ± 0.678 cm), compared to the control (34.1 ± 1.50 cm). Conversely, the lowest shoot length among all crops was in *O. sativa* control plots (8.2 ± 1.49 cm), with the 10 t/ha treatment (10.5 ± 1.37 cm) and the 15 t/ha treatment (9.34 ± 1.43 cm) (Fig 2b; Table 3).

#### Number of leaves.

Biochar treatment showed an increase in leaf count across the studied crops (Fig 2c; Table 2). The ANOVA results showed a significant effect of treatment on leaf count (F = 8.318, p < 0.001), with a significant interaction between treatment and crop (F = 5.136, p < 0.001) (Table 2). The highest leaf count was recorded for *C. annuum* under the 10 t/h treatment (17.6 ± 0.245) and *S. lycopersicum* under the 10 t/h treatment (8.6 ± 0.245), whereas the lowest counts were observed in *S. melongena* under the 15 t/h treatment (3.4 ± 0.245) (Fig 2c). Significant differences were found in leaf count for *C. annuum* between control (16.6 ± 0.245) and 10 t/h treatments (17.6 ± 0.245) (p < 0.05) and for *S. lycopersicum* between control (7.4 ± 0.245) and 10 t/h treatments (8.6 ± 0.245) (p < 0.01) (Table 3).

#### Root to shoot weight ratio.

The effect of biochar on the root to shoot weight ratio varied significantly depending on the crop variation in our study (Fig 2d; Table 2). The ANOVA results demonstrated a significant effect of treatment on the root to shoot weight ratio (F = 8.764, p < 0.001) and a significant interaction between treatment and crop (F = 6.532, p < 0.001) (Table 2). The highest ratios were observed in *O. sativa* control plots (2.18 ± 0.244) and *T. aestivum* control plots (1.02 ± 0.154), while the lowest ratio was found in *C. annuum* under the 15 t/h treatment (0.324 ± 0.0219) (Fig 2d). Significant differences in the root shoot weight ratio were observed for *O. sativa* between control (2.18 ± 0.244) and both 10 t/h (0.927 ± 0.206) (p < 0.001) and 15 t/h treatments (0.889 ± 0.164) (p < 0.001), as well as for *S. lycopersicum* between control (0.292 ± 0.0834) and 15 t/h treatments (0.799 ± 0.126) (p < 0.05) (Table 3).

### Biochar effects on soil nutrient

**Fig 3:**
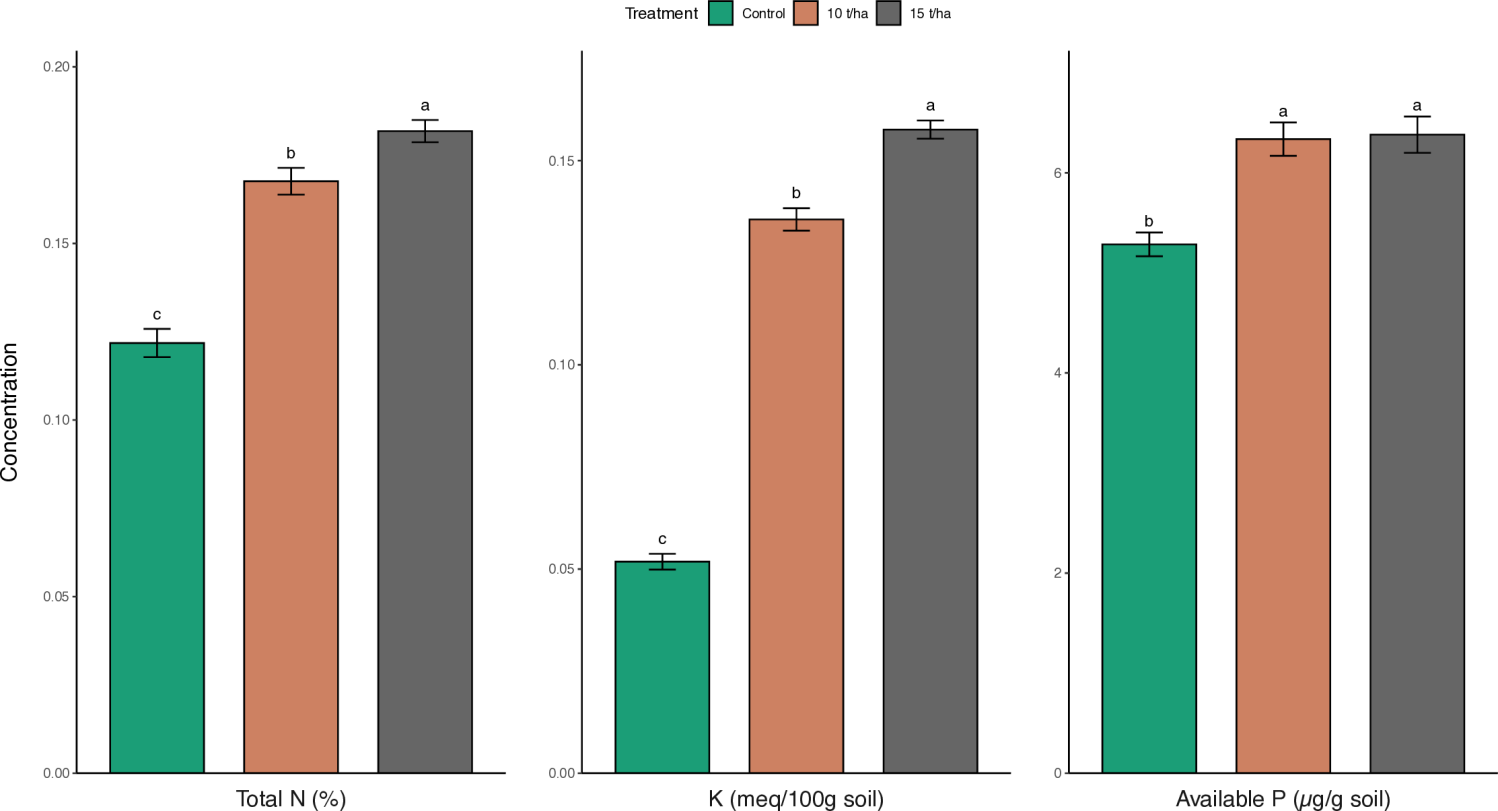
Bar plot illustrating soil concentrations of Total N (%), K (meq/100g soil), and available P (µg/g soil) under control, 10 t/ha biochar dose, and 15 t/ha biochar dose.

Biochar treatment significantly impacted (p < 0.001) soil total nitrogen (N) (%), potassium (K) (meq/100g), and available phosphorus (P) (µg/g soil) in this experiment (Fig 3).

Control soil had a total N concentration of 0.122 ± 0.004% (mean±SE), while soil treated with 10 t/ha biochar showed a significantly higher N concentration of 0.168 ± 0.0038% (p < 0.001). The 15 t/ha biochar treatment further increased soil N to 0.182 ± 0.00315%, significantly higher than the 10 t/ha treatment (p < 0.05) (Fig 3). Similarly, control soil potassium was 0.0518 ± 0.00193 meq/100g, whereas 10 t/ha biochar treatment increased soil K to 0.136 ± 0.00275 meq/100g (p < 0.001). The 15 t/ha biochar treatment further increased soil K to 0.158 ± 0.00223 meq/100g, significantly higher than the 10 t/ha treatment (p < 0.001). For available phosphorus, control soil measured 5.28 ± 0.118 µg/g. The 10 t/ha biochar treatment increased available P to 6.34 ± 0.167 µg/g (p < 0.01). There was no significant difference in available phosphorus between the 10 t/ha and 15 t/ha biochar treatments, with the 15 t/ha treatment measuring 6.38 ± 0.181 µg/g (p > 0.05) (Fig 3).

## Discussion

### Effects of Biochar on seed germination

Early germination is essential as it enables crops to rapidly establish and utilize favorable growing conditions before the onset of unfavorable weather or nutrient depletion [29]. Additionally, fast germination reduces the exposure period to pests and diseases, thereby improving crop yield and resilience [30]. Our study found that the seed germination (%) of different crops was significantly affected by biochar. This result is consistent with earlier research suggesting that biochar may improve overall germination potential [31,32]. In particular, our findings showed that the germination rates of *Oryza sativa* (rice) and *Capsicum annuum* (chili) significantly increased when biochar was applied. However, seed germination is inherently influenced by crop variety and genetic factors. While the results demonstrated a positive effect of biochar, the observed variations among crops should be interpreted within the context of species-specific germination traits. To mitigate the confounding effects of varietal differences, future studies should incorporate multiple cultivars within each species to ensure a more comprehensive assessment of biochar’s efficacy.

Additionally, biochar’s influence on seed germination and seedling growth was not uniform across all conditions, as its effects also varied with soil properties. Moreover, in our study, the use of biochar significantly influenced the early germination rate, which was assessed after 7 days. In particular, the early germination rate for *C. annuum* and *O. sativa* was notably higher when treated with biochar. Biochar directly influences seed germination through several mechanisms.

The application of biochar increased soil capillary water-holding capacity and non-capillary porosity, enhancing effective soil moisture and aeration [33]. This improvement in soil conditions promoted seed germination. Furthermore, a previous study demonstrated that biochar could produce ethylene independent of soil or microbial inoculation. Ethylene plays a crucial role in seed germination and the release of dormancy in various species [34].

### Biochar effects on early growth parameters

The impact of biochar on root length varied among different test crops used in the experiment, with significant increases observed in *C. annuum* and *O. sativa*. Previous studies have suggested that biochar can promote root growth by enhancing soil structure and increasing the availability of essential nutrients such as phosphorus, which is present in higher concentrations in our woody biochar (Table 2) [35]. The increased root length observed in our study supports the hypothesis that biochar can create a more conducive rooting environment, potentially leading to improved nutrient uptake and plant growth. The application of biochar had a significant impact on the length of the shoots, especially in *S. lycopersicum*, where the 10t/h treatment resulted in the longest shoots. This observation aligns with the findings of Guo et al., (2021) [17], who attributed the effects of biochar to its enhancement of soil capillary water-holding capacity and increased availability of phosphorus. Improved soil capillary water, which constitutes effective moisture, is transported to plant roots through capillary action and directly absorbed by the plants, thus promoting shoot growth. Additionally, the moisture-retentive properties of biochar ensure a consistent water supply during critical growth stages, further contributing to increased shoot length.

The increase in leaf count observed in most crops with biochar treatment indicates improved overall plant health and vigor. Previous research has also found similar results, showing that biochar amendments led to higher leaf biomass due to enhanced nutrient availability and soil moisture retention. This process, occurring through the xylem stream, enhances nitrogen accumulation inside plant cells, leading to a higher number of branches and leaves [36,37]. The higher leaf count in *C. annuum* and *S. lycopersicum* suggests that biochar can promote more robust vegetative growth, potentially leading to higher photosynthetic capacity and greater yield [38]. Although biochar had a significant effect on leaf production, leaf expansion and branching patterns are inherently regulated by genetic factors. Therefore, these findings should be interpreted within the framework of species-specific physiological responses. Further investigations incorporating multiple cultivars are necessary to evaluate the consistency of these effects across different genetic backgrounds.

The ratio of root weight to shoot weight varied significantly among different crops, with noticeable decreases seen in *C. annuum*. This change in ratio is likely due to the way plants allocate biomass to their roots and shoots in response to improved soil conditions resulting from the use of biochar [39]. A lower root-to-shoot weight ratio in plants treated with biochar may indicate that investment of less energy is needed for root growth as the improved soil fertility and structure facilitate easier access to nutrients and water around the roots, allowing more resources to be directed to shoot growth and reproduction.

### Potentials for future sustainable crop management

This study highlights the significant potential of biochar for advancing sustainable crop management in densely populated regions like Bangladesh. By focusing on highly cultivated tropical crops, our research underscores the role of biochar in cost-effectively optimizing agricultural productivity while minimizing environmental impacts. It can significantly reduce the environmental impacts while improve soil fertility. Soil is the life-sustaining source; however, soil health is drastically affected by intensive agricultural inputs like chemical fertilizers and pesticides. Biochar is considered the best organic source to act as a soil amendment due to its stability and ability to retain nutrients that reduce leaching [40,41]. Our findings showed that biochar application notably improved seed germination and early growth metrics across selected crops such as *C. annuum* and *O. sativa*, attributable to its enhancements in soil organic matter, structure, and water retention [42]. This improvement is crucial for maximizing crop yields [43]. To feed the ever-increasing population, agricultural sciences have always been a top priority for nations seeking new innovations and technologies to boost crop production.

Incorporating biochar into agronomic practices as a soil amendment process is one of the best efforts to increase food production with reduced reliance on synthetic fertilizers, thus lowering associated carbon footprints. In Bangladesh, where efficient land use and cost-effective production methods are paramount, biochar presents a viable solution for sustainably enhancing soil fertility and productivity. Additionally, biochar has a high surface area and porous structure that allows it to adsorb and retain nutrients. This helps to reduce nutrient leaching while making nutrients more available to plants over time [44]. The ability of biochar to retain nutrients is especially beneficial in sandy or degraded soils where nutrient leaching is a major concern like Bangladesh [20].

Recently, biochar has been extensively used as the carrier of slow-release fertilizers loaded with different macro and micronutrients. While these findings underscore the potential benefits of biochar, further research is required to evaluate its effects across diverse crop varieties under varying biochar formulations and application rates. Such investigations will be essential for refining recommendations to support its large-scale implementation in agricultural systems.

Our study is a preliminary step in introducing biochar in the agricultural field of Bangladesh, either as a sole amendment or as a slow-release fertilizer in biochar pellets. Overall, our findings align with the broader objective of integrating low-cost, low-impact practices into agriculture, supporting the transition toward more sustainable and resilient cropping systems. Future research should extend these insights through long-term field trials and diverse crop evaluations to fully leverage biochar’s benefits in tropical and densely populated agricultural settings.

## Conclusion

This study demonstrates the potential of biochar as an effective soil amendment for improving seed germination rate and early growth parameters in crops. The response to biochar application was species-specific, with *O. sativa* and *C. annuum* showing enhanced germination rates and increased root lengths. The impact of biochar varied depending on the plant function, such as germination, root growth, and shoot growth, which were not uniformly affected across species. Biochar application also improved shoot length and leaf count, indicating better plant health and vigor. These benefits could be attributed to enhanced microbial activity, increased nutrient availability, and improved soil moisture retention. The findings suggest that the effectiveness of biochar depends on the specific crop and growth parameter. Future research should focus on long-term field trials and explore the effects of biochar on a broader range of crops. Investigating optimal application rates and biochar types for different agricultural systems will further enhance understanding and practical application. The results underscore potential of biochar in improving soil structure, nutrient availability, and water retention, contributing to sustainable agricultural practices and increased crop yields. The findings indicate that biochar can inform agricultural policies aimed at soil conservation and sustainability. Its integration into farming strategies could enhance resilience, reduce chemical fertilizer reliance, and improve food security in regions impacted by soil degradation and climate change.

## Supporting information

S1 File Detailed properties of the biochar used in this experiment, including physicochemical characteristics and elemental composition (DOCX)
