# Supplementary material for: Biochar enhances seed germination and crop early growth for sustainable agriculture in Bangladesh
Source: PLoS One. 2025 Mar 18;20(3):e0320005. doi: 10.1371/journal.pone.0320005 (PMC11918420; doi:10.1371/journal.pone.0320005)
Supplement: S1 File — (DOCX) [file pone.0320005.s001.docx]

**SUPPLEMENTARY MATERIALS**

**Supplementary 1:** Physicochemical properties of the kiln-derived *Acacia* *auriculiformis* wood biochar used in this experiment. Previously characterized in Karim et al. (2020) as:

| Properties | Unit | Mean ± SE (n = 3) |
| --- | --- | --- |
| Al | % | 0.083 ± 0.003 |
| As | ppm | 1.000 ± 0.000 |
| Ba | ppm | 17.667 ± 0.333 |
| Total C | % | 72.1 ± 0.073 |
| Ca | % | 1.820 ± 0.031 |
| Cd | ppm | 0.150 ± 0.041 |
| Ce | ppm | 1.000 ± 0.000 |
| Co | ppm | 0.467 ± 0.033 |
| Cr | ppm | 11.000 ± 2.517 |
| Cu | ppm | 16.200 ± 0.208 |
| Fe | % | 0.900 ± 0.015 |
| K | % | 2.060 ± 0.038 |
| La | ppm | 0.533 ± 0.033 |
| Li | ppm | 0.433 ± 0.033 |
| Total N | % | 1.8 ± 0.014 |
| Na | % | 0.051 ± 0.002 |
| Nb | ppm | 1.067 ± 0.033 |
| Ni | ppm | 4.333 ± 0.133 |
| P | % | 0.235 ± 0.002 |
| Rb | ppm | 25.000 ± 0.404 |
| Pb | ppm | 3.167 ± 0.067 |
| Mg | % | 0.120 ± 0.000 |
| Mn | ppm | 131.667 ± 2.728 |
| Mo | ppm | 1.767 ± 0.033 |
| Sb | ppm | 0.100 ± 0.000 |
| Sn | ppm | 1.467 ± 0.088 |
| Sr | ppm | 70.333 ± 1.453 |
| Th | ppm | 0.133 ± 0.033 |
| Tl | ppm | < 0.05 |
| W | ppm | 0.200 ± 0.000 |
| Y | ppm | 0.333 ± 0.033 |
| Zn | ppm | 799.667 ± 35.751 |
| Zr | ppm | 2.233 ± 0.393 |
| Volatile matter | % | 70.2 ± 0.89 |
| Ash content | % | 7.9 ± 0.12 |
| pH | – | 7.6 ± 0.11 |
| EC | μS.cm^−1^ | 532.3 ± 17.6 |
| Bulk density | g·cm^−3^ | 0.141 ± 0.005 |
